# Whole-genome sequencing surveillance of Siberian tick-borne encephalitis virus (TBEV) identifies an additional lineage in Kyrgyzstan

**DOI:** 10.1016/j.virusres.2024.199517

**Published:** 2024-12-22

**Authors:** Jake D'Addiego, Mollie Curran-French, Jack Smith, Asankadyr T Junushov, Irena Breininger, Barry Atkinson, John Hay, Roger Hewson

**Affiliations:** a UK Health Security Agency, Science Group, Porton Down, Salisbury, UK; b Department of Infection Biology, Faculty of Infectious and Tropical Diseases, London School of Hygiene and Tropical Medicine, London, UK; c Health Protection Research Unit (HPRU) in Emerging and Zoonotic Infections, Institute of Infection and Veterinary and Ecological Science, University of Liverpool, Liverpool, UK; d National Academy of Sciences of the Kyrgyz Republic, Bishkek, Kyrgyz Republic; e Ministry of Healthcare of the Kyrgyz Republic, Bishkek, Kyrgyz Republic; f State University of New York, Buffalo, NY, USA

**Keywords:** TBEV, Whole-genome sequencing, Molecular surveillance, Targeted enrichment, Tick-borne viruses

## Abstract

Tick-borne encephalitis virus (TBEV) is the most prevalent tick-borne viral disease in Europe and Asia. There are three main subtypes of the virus: European, Siberian, and Far Eastern, each of which having distinctive ecology, clinical presentation, and geographic distribution. In recent years, other TBEV subtypes have been described, namely the Himalayan and Baikalian subtypes. Differences in virulence between TBEV subtypes have been described, with the Far Eastern subtype causing the most severe disease in humans. Considering the emergence of new TBEV foci, the genetic characterisation of the virus in endemic regions is crucial to not only better understand its epidemiology, but also to identify possible genetic determinants of virulence, as well as develop accurate diagnostics and therapeutics.

In our previous study, we identified TBEV in six localities of the Kyrgyz Republic (Kyrgyzstan), and Ala-Archa National Nature Park as a focus of TBEV transmission. Whilst we were able to retrieve the first partial TBEV sequence from Kyrgyzstan from *Ixodes persulcatus* ticks, we were unable to retrieve a complete genome sequence at that time.

In this study, we have utilised a sequence-independent single-primer amplification (SISPA) protocol and retrieved the complete genome sequence of our previous 2009 TBEV tick sample (strain KY09) producing the third complete TBEV genome from Kyrgyzstan, and the first genome from the region clustering within the Vasilchenko lineage, suggesting a wider distribution for the lineage than was previously thought.

We have also developed a tiling amplicon scheme for Siberian TBEV (TBEV-Sib) which produced > 90 % reference coverage at 100x sequencing depths for samples with as little as 1.13×10^4^ RNA copies/ml. Since high viral loads are rare in TBEV clinical samples, the developed protocol adds value to TBEV-Sib endemic regions by offering a novel set of primers to further amplify the viral genome prior to sequencing.

## Highlights

• A historical TBEV genome has been recovered from *Ixodes persulcatus* ticks in Kyrgyzstan.
• Phylogenetic analysis revealed the presence of the Vasilchenko lineage of TBEV-Sib in Kyrgyzstan.
• A novel, tiling PCR scheme for TBEV-Sib was developed, which produced near complete genome coverages at 100x sequencing depths from samples with as little as 1.13×10^4^ RNA copies/ml.

## 1 Introduction

Tick-borne encephalitis virus (TBEV) is an orthoflavivirus within the *Flaviviridae* family (species *Orthoflavivirus encephalitidis*). TBEV is the most prevalent tick-borne viral disease in Eurasia, causing thousands of recorded human infections each year (Walter et al., 2020; O Donoso et al., 2008). The clinical manifestations of TBEV infection can range from non-specific, flu-like symptoms to severe neurological complications such as meningitis, encephalitis, and long-term cognitive impairments (Dabas et al., 2023).

There are three main subtypes of the virus: European (TBEV-Eu), Siberian (TBEV-Sib) and Far-Eastern (TBEV-FE) each of which having distinctive ecology, clinical presentation, and geographic distribution. More recently, other subtypes of TBEV have been described, namely the Himalayan (TBEV-Him) (Dai et al., 2018) and Baikalian (TBEV-Bkl) (Kovalev and Mukhacheva, 2017) subtypes, the latter further divided into two genotypes, TBEV-Bkl-1 and TBEV-Bkl-2 (Deviatkin et al., 2020). The TBEV-Sib subtype is the most genetically heterogeneous, and previous studies attempted to classify different TBEV-Sib lineages based on amino acid signatures within the glycoprotein E sequence (Kovalev and Mukhacheva, 2017; Forghani et al., 2023). Currently, the Siberian subtype is divided into five different lineages: Zausaev, Vasilchenko, Baltic, Obskaya and Bosnia (Kutschera and Wolfinger, 2022). Differences in virulence between different TBEV subtypes have been observed, with TBEV-FE causing the most severe disease with a case fatality rate ranging between 5 and 20 % (Dumpis et al., 1999), TBEV-Sib associated with a less severe disease with a case fatality rate ranging between 1.8–3 % (Poponnikova, 2006) and TBEV-Eu with a case fatality rate below 1 % (Kutschera and Wolfinger, 2022; Beaute et al., 2018).

The virus contains a positive-sense, single-stranded RNA genome of about 11 kb in length (Pustijanac et al., 2023), which is translated as a polyprotein and subsequently cleaved into three structural (C, prM and E) and seven non-structural (NS) proteins (NS1, NS2A, NS2B, NS3, NS4A, NS4B and NS5) (Tkachev et al., 2020). The genome is flanked by two highly structured untranslated regions (UTRs), a smaller 5′-end containing a type-I cap, and a longer 3′-end of variable length which may or may not contain an internal poly-A tail (Pustijanac et al., 2023). Asghar et al. (2016) have shown that a longer poly-A tail is associated with quasispecies emergence, decreased replication rate and increased neuroinvasiveness and neurovirulence in mice. Secondary structures in the non-coding regions participate as cis-regulatory elements in genome replication, translation and viral particle assembly (Gritsun et al., 2003).

The main vectors of the virus are *Ixodes ricinus* ticks for the European subtype and *I. persulcatus* and *Ixodes ovatus* ticks for the Siberian and Far Eastern subtypes respectively (Worku, 2023), however at least 22 other tick species belonging to the genera *Dermacentor, Hyalomma* and *Rhipicephalus* as well as other *Ixodes* species have also been identified as competent vectors for the virus (Pustijanac et al., 2023). TBEV is transmitted to susceptible reservoirs species including numerous small mammals such as rodents, and larger mammals including ungulates through the bite of an infected tick. In addition to tick bite, TBEV can be transmitted to humans through consumption of unpasteurised milk and milk products (Lindquist and Vapalahti, 2008), with rare cases of transmission to infants from mothers through breastfeeding (Dabas et al., 2023) having been observed.

The virus is endemic in several European countries, with the highest number of cases reported in Czechia, Germany and Lithuania, although new TBEV foci are being detected including in the UK (Holding et al., 2020). In Asia, the virus is widespread in Russia, Kazakhstan, Kyrgyzstan, Mongolia, China, Korea and Japan (Kwasnik et al., 2023). There are currently only 354 complete TBEV genomes published on the Bacterial and Viral Bioinformatics Resource Center (July 2024) of which 47 % are from Russia, with the rest of the Asian countries representing 8.8 % of the sequences combined. The genetic characterisation of TBEV in endemic regions is of paramount importance to not only better understand its epidemiology, but also to identify possible genetic determinants of virulence, as well as develop accurate diagnostics and therapeutics.

Our previous study carried out in 2009 identified TBEV in six different localities of the Kyrgyz Republic (Kyrgyzstan), and Ala-Archa National Nature Park as a focus of TBEV transmission (Briggs et al., 2011). Whilst we were able to retrieve the first partial TBEV sequence from Kyrgyzstan (HM641235) which was representative of sequences collected from *I. persulcatus* tick pools as well as liver samples from the Ward's field mouse *Apodemus pallipes*, we were unable to retrieve a complete genome sequence at that time. There are currently only two published complete genome sequences of TBEV from what is today known as Kyrgyzstan. The first TBEV genome sequence from Kyrgyzstan was deposited in 2014 (KJ626343) and recovered from a 1986 tick isolate collected near Issyk-Kul lake (former USSR), while the second genome was recovered from ticks collected from cattle in the Kemin region in May 2023 (OR896869) and was found to be most closely related to the Bosnia strain MH645616 by phylogenetic analysis (Jung et al., 2023).

In this study, we have utilised a sequence-independent single-primer amplification (SISPA) protocol and retrieved the complete genome sequence of our previous 2009 TBEV tick sample (strain KY09), producing the third complete TBEV genome from Kyrgyzstan and the first genome from the region clustering within the Vasilchenko lineage.

Furthermore, we have developed and tested a targeted, tiling amplicon scheme to enrich for TBEV-Sib sequences with which we were able to retrieve greater than 90 % of the genome with 100x sequencing depth in samples with as little as 1.13×10^4^ copies/ml. Comparative studies between amplicon-based sequencing versus metagenomic approaches on clinical samples have highlighted the superiority of a targeted approach to recover near complete genome sequences from samples with low virus titres (Zakotnik et al., 2022; D'Addiego et al., 2024). Since a high viral load is rare in TBEV infected patients (Saksida et al., 2018), the developed protocol adds value to TBEV endemic regions with circulating TBEV-Sib by offering a novel set of primers to further amplify the viral genome prior to sequencing.

## 2 Material and methods

### 2.1 Virus inactivation and RNA extraction

One hundred microlitres (µl) of a tick pool homogenate in RNALater (Thermo Fisher Scientific) was inactivated using 300 µl of RLT buffer (QIAGEN) with the addition of 300 µl 70 % molecular grade ethanol after 10 min within a Containment Level (CL) 3 facility. Inactivated sample was then transferred to a CL2 facility for RNA extraction. Extraction was performed using the RNEasy Mini kit (QIAGEN) following the manufacturer's RNA Cleanup protocol.

### 2.2 RNA quantification

Primers and probes for quantitative real-time reverse transcription polymerase chain reaction (RT-PCR) were derived from Schwaiger and Cassinotti (2003). Five microlitres of the extracted RNA was amplified in 20 µl reactions containing 500 nM of forward primer, 500 nM of reverse primer, 250 nM of BHQ probe and 1x TaqMan™ Fast Virus 1-Step Master Mix (Thermo Fisher Scientific).

Copy number of TBEV genomic RNA were determined based on standard curves of a synthetic RNA *in-vitro* transcript (IVT) designed against a TBEV-Eu reference (U27495.1). RT-PCR was performed on a QuantStudio 7 Flex Real-Time PCR System (Applied Biosystems) platform with the following cycling parameters: 50 °C for 10 min, 95 °C for 2 min and 45 cycles of 95 °C for 20 s and 60 °C for 40 s.

### 2.3 Sequencing library preparation

SISPA enrichment was carried out as previously described (D'Addiego et al., 2024). Illumina sequencing libraries were prepared utilising a Nextera XT V2 kit (Cat. No. FC-131–1096; Illumina) and sequenced on a 2 × 150-bp paired-end Illumina MiSeq instrument.

The cDNA input for the tiling amplicon scheme from extracted RNA was synthesised using SuperScript™ IV First-Strand Synthesis System (Thermo Fisher Scientific, cat. 18,091,050) in final 20 µl reactions as previously described (D'Addiego et al., 2024).

Target enrichment was based on a previously published protocol (Quick et al., 2017) using the Primal Scheme primer designer software. Two separate primer pools were prepared, each containing primers targeting alternate fragments. Primers were designed against TBEV-Sib reference sequences (MH645616, KP345889, MN520112). Primer sequences are provided in Supplementary Table S1.

Details of each primer pool with final primers concentrations are provided in Supplementary Table S1. 2.5 µl of cDNA was amplified in triplicate 25 µl reactions containing either one of the two sets of primer pools utilising Q5 High-Fidelity 2X Master Mix (NEB, cat. no. M0492 L). Master-mix composition is provided in Supplementary Table S1. cDNA was amplified in a thermocycler with the following cycling parameters: 98 °C for 30 s, 30 cycles of 98 °C for 15 s, 57 °C for 30 s and 70 °C for 30 s and held at 4 °C. PCR amplicons were cleaned up with AMPure XP Reagent (Beckman Coulter, cat. no. A63881) and stored at −20 °C.

Barcoded nanopore sequencing libraries were prepared from 65 ng of cDNA with the Ligation Sequencing kit SQK-LSK-110 and Native Barcoding Kit EXP-NBD196 (ONT) as described previously (D'Addiego et al., 2024). Libraries were sequenced for 24 h on a FLO-MIN106D flow cell using a Mk1C MinION device (Oxford Nanopore Technologies).

### 2.4 Bioinformatics and phylogenetic analysis

Illumina reads were mapped against a reference genome (MN520112) utilising BWA MEM with default settings (Li and Durbin, 2009). Nanopore sequencing reads were filtered against the reference genome and based on length (0.4–0.6 Kb) and quality (minimum 8) using Filtlong software. Nanopore sequencing reads were mapped against the reference utilising minimap2 (Li, 2018). Reference coverage and sequencing depth statistics were derived utilising SAMtools (Danecek et al., 2021) depth, coverage and flagstat functions.

Consensus sequences from metagenomic data were derived from sorted BAM files utilising BCFtools (Danecek et al., 2021), masking regions with coverage below 10x. For targeted enrichment data, primer sequences were first removed from sorted BAM files with BAMClipper (Au et al., 2017) prior to consensus generation with BCFtools as described above (regions with coverage below 1x were masked for nanopore sequencing data). Generated Illumina genome from TBEV strain KY09 was deposited on GenBank (PQ015165).

A Maximum Likelihood (ML) phylogeny was generated with MEGA (Kumar et al., 2016) (version 7.0.26) applying the general time reversible model. Sequences were aligned using MUSCLE (Edgar, 2004). Alignments were trimmed to exclude low coverage regions to a final length of 10,245 bp. Bootstrap support values were generated with 1000 replicates.

## 3 Results

### 3.1 Greater than 90 % genome coverage at 100x sequencing depth was produced with a tiling amplicon scheme for samples with low viraemia

**Fig. 1:**
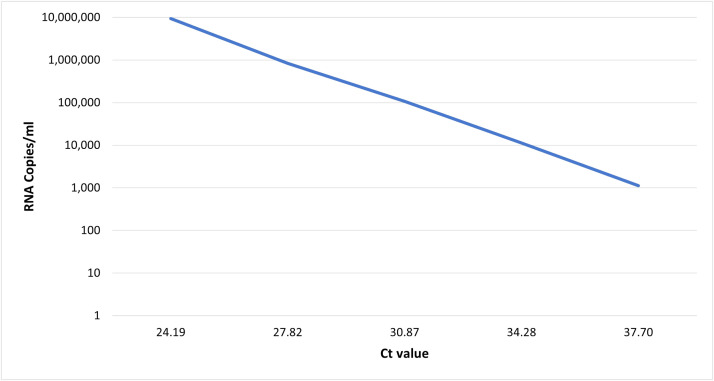
RNA copies/ml over Ct value for the serially diluted tick RNA samples.

RNA copies/ml in the serially diluted tick RNA ranged between 1.13×10^3^ to 9.42×10^6^, with corresponding Ct values ranging from 37.70 to 24.19 (Fig. 1).

**Fig. 2:**
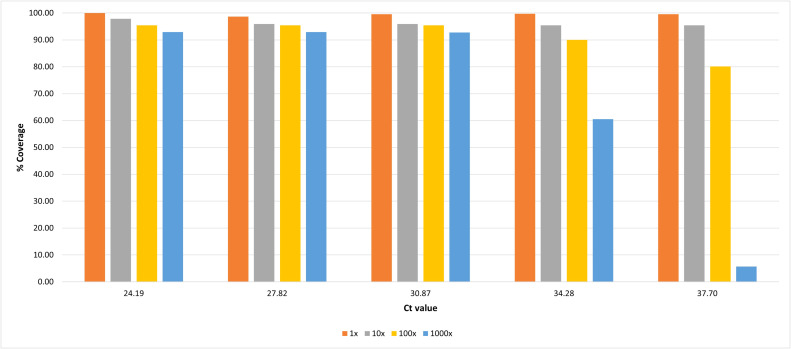
Reference coverage (%) and sequencing depths at 1x (orange), 10x (grey), 100x (yellow) and 1000x (blue) for the serially diluted RNA.

Reference coverages ranged between 98.65 %−100 % at 1x sequencing depth, 95.40 %−97.85 % at 10x depth, 82.58 %−95.44 % at 100x depth and 5.68 %−92.94 % at 1000x depth (Fig. 2).

### 3.2 On average, the positive samples produced sequencing depths averages ranging from 439.63x to 30,781.27x

**Fig. 3:**
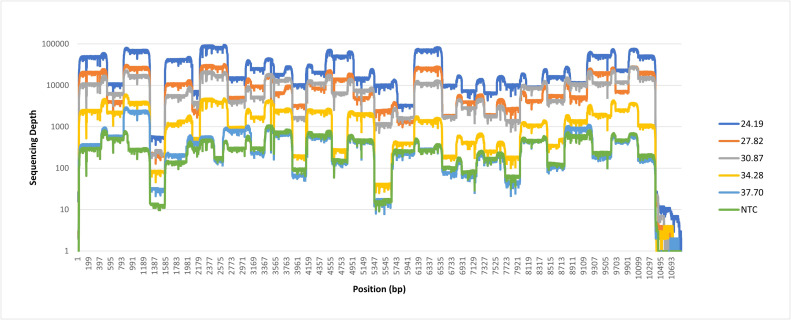
Sequencing depths across the TBEV reference genome.

The RNA samples produced average sequencing depths ranging from 439.63x to 30,781.27x. The negative template control (NTC)’s average sequencing depth was 325.95x (Fig. 3).

**Fig. 4:**
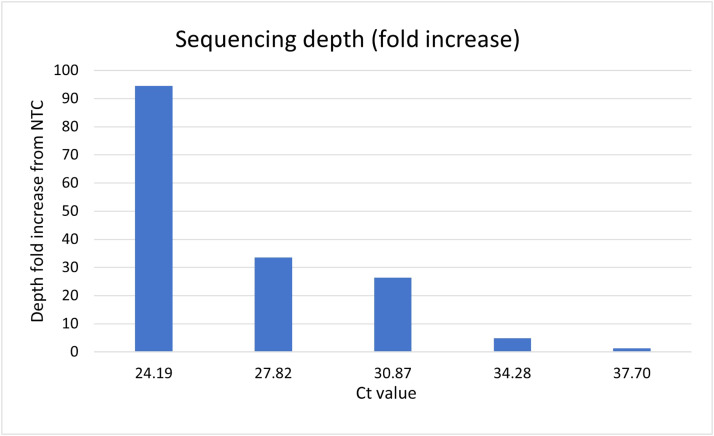
Fold-increase in average sequencing depth between RNA samples and negative template control (NTC).

Average sequencing depth fold increases of 94.44, 33.57, 26.33, 4.81 and 1.35 were achieved for samples with Ct values of 24.19, 27.82, 30.87, 34.28 and 37.70 respectively compared to the negative template control (NTC) (Fig. 4).

### 3.3 Complete genome phylogenetic analysis reveals the presence of at least two separate TBEV-Sib lineages in Kyrgyzstan

**Fig. 5:**
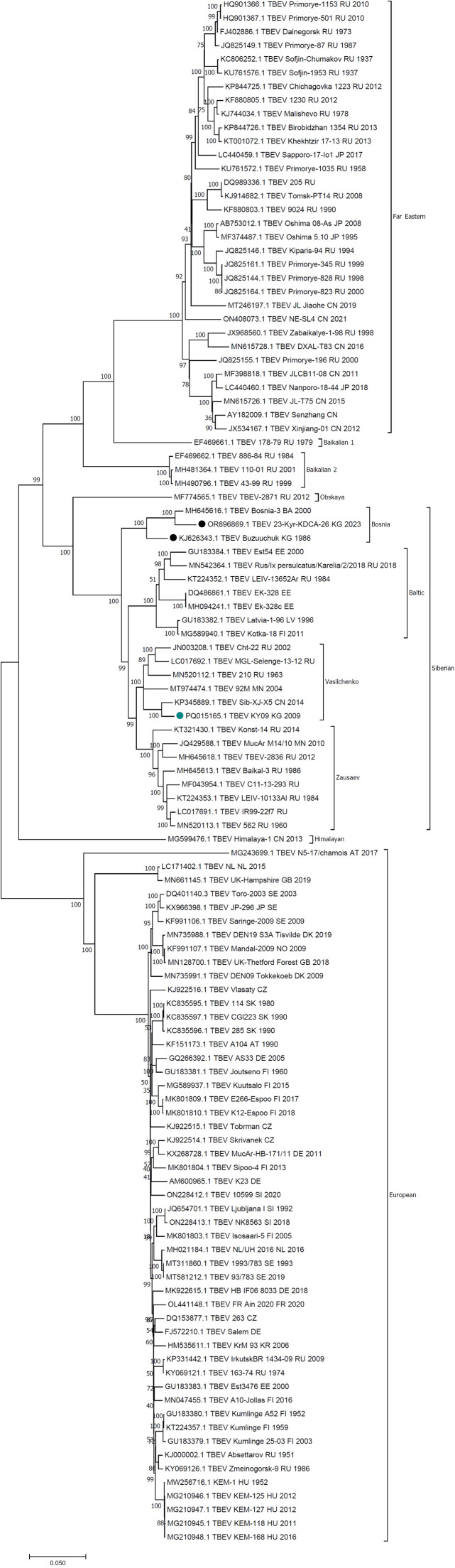
Maximum Likelihood (ML) phylogeny of complete TBEV genome sequences. Previously published TBEV genome sequences from Kyrgyzstan are indicated by the black circles. The newly generated genome sequence (PQ015165) is indicated by the teil-coloured circle.

Complete genome phylogenetic analysis of the tick TBEV isolate reveals its affiliation within the Vasilchenko lineage of the Siberian subtype of TBEV, with closest complete genome being KP345889 (Fig. 5).

Amino acid signatures in the glycoprotein E sequences (Table 1) suggest affiliation of the KY09 tick strain (PQ015165) and OR896869 to clusteron 3A/3A^2^ and 3L/3L^2^ (TBEV-Sib^Asia^/TBEV-Sib^S.-Sib^), and KJ626343 to clusteron 3 G (TBEV-Sib^Balt^).

**Table 1:** TBEV clusteron amino acid signatures for the published genomes from Kyrgyzstan.

GenBank Acc. No.	Clusteron	Clusteron specific amino acid signature																		
		119	120	122	126	128	136	151	165	167	175	178	188	192	195	204	221	228	232	234
**PQ015165**	**3A/3A^2^**	A	A	E	K	T	K	V	F	V	T	D	V	V	A	K	N	K	A	H
**OR896869**	**3L/3L^2^**	A	A	E	K	I	K	V	F	V	T	D	V	V	A	K	N	K	A	H
**KJ626343**	**3G**	V	A	E	K	T	K	V	F	V	N	D	V	V	A	K	N	K	A	H

*Positions are given for the glycoprotein E amino acid sequence.

## 4 Discussion

Although TBEV remains the most prevalent tick-borne viral disease in Europe and Asia, with new emerging foci having been described in recent years, little is still known about the viral genetic determinants of virulence. Considering the high conservation rate in the amino acid (AA) sequence of the polyprotein, studies have suggested that determinants of virulence in TBEVs may be represented by a minority of AA changes (Pulkkinen et al., 2018). While the TBEV-FE subtype is associated with the highest mortality and virulence, within subtype differences in virulence have also been observed. For instance, *in vivo* and *ex vivo* studies have shown that the Hypr strain is more virulent than the Neudörfl strain within the TBEV-Eu subtype (Hoornweg et al., 2023; Mandl et al., 1997), and viruses related to the Sofjin and Senzhang strains cause more severe disease in humans than strains related to the Oshima strain within the TBEV-FE subtype (Tkachev et al., 2020).

These studies highlight the importance of the continued genetic characterisation of TBEV viruses in both, the tick vectors and in clinical settings. This ongoing research is crucial not only for development and optimisation of molecular diagnostics, but also for identifying possible determinants of virulence. Such findings also provide important information to aid in the development of effective therapeutics and vaccines.

Several studies have reported the limitations of metagenomic sequencing on samples with low viraemia (Zakotnik et al., 2022; D'Addiego et al., 2024). Zakotnik et al. have recovered TBEV-Eu genomes by metagenomic approaches only from samples with viral loads above 10^4^ copies/ml. Similarly in our previous study on Crimean-Congo haemorrhagic fever virus (CCHFV), the threshold for successful near-complete genome recovery with non-targeted approaches was even higher at approximately 10^6^ copies/ml (Ct 27). Saksida et al. (2018) found that the copies of TBEV RNA in clinical samples ranged from 10^3^ to 10^6^ copies/ml, with an average of 4.47×10^4^ copies RNA/mL, which is at the threshold for successful complete genome recovery with non-targeted sequencing approaches. These findings underscore the importance of developing targeted approaches for successful whole genome recovery.

In this study, we have developed and tested the first TBEV-Sib tiling amplicon scheme on a serial dilution of tick TBEV RNA (Fig. 1) covering the range of reported viral loads for TBEV in clinical samples. Our results showed that we were able to achieve over 90 % reference coverage and 1000x sequencing depths from samples with 1.07×10^5^ RNA copies/ml, and 100x sequencing depths from samples with 1.13×10^4^ RNA copies/ml (Fig. 2).

Whilst we did recover a near complete genome sequence from our sample with 1.13×10^3^ copies/ml at a sequencing depth of 10x, we also recovered a complete genome with similar sequencing depth from our negative template control (NTC) (Fig. 3). Furthermore, the similar sequencing depths observed for both sets of primer pools for this sample as well as the NTC (Fig. S1.E, F) does not suggest true amplification of alternate genome fragments like observed for the higher dilutions (Fig. S1.A–D). This indicates contamination during the sequencing library preparation and suggests that studies utilising targeted PCR-based enrichment strategies, which can produce highly concentrated copies of the target products, should be cautious of low depth signals from samples with low viraemia (below 10^3^ copies/ml). Such samples may need to be processed separately from concentrated samples to avoid cross contamination, and barcoding reactions using different primer pools separately may aid in distinguishing true positive signals from low coverage contamination. Our sample with 1.13×10^4^ copies/ml produced near complete reference coverage (>90 %) at 100x sequencing depth with an average sequencing depth 4.81-fold greater than the NTC (Figs. 4 and S1.D), nonetheless sequencing results should be interpreted with caution below this threshold, particularly at sequencing depths of 10x or below.

As in the study by Zakotnik et al. (2022), who produced a tiling amplicon scheme for the European subtype of TBEV, we have also recovered 100 % of the polyprotein sequence from the tick-derived TBEV-Sib strain KY09 with our newly developed amplicon scheme, as well as a complete 5′ UTR. However a complete 3′ UTR was only recovered from our highest viral load dilution (9.42×10^6^ copies/ml), likely due to the complex secondary structures which are present in this region of the TBEV genome (Gritsun et al., 2003) affecting PCR efficiency.

Kovalev and Mukhacheva (2017) have developed a typing scheme for TBEV-Sib which groups TBEV-Sib into clusterons based on amino acid signatures in the glycoprotein E sequence, and first described the Baikalian lineage as a distinct lineage within the TBEV-Sib subtype. However the availability of complete genome sequencing data has re-defined the evolutionary relationships within the Siberian subtype which now includes five distinct lineages (Fig. 5). While the glycoprotein E sequence suggest affiliation of the tick genome to clusteron 3A/3A^2^ (Asian Siberian or South Siberian group), phylogenetic analysis of the complete genome sequence confirmed affiliation of the tick genome within the Vasilchenko lineage (or the East Siberia group with the clusteron grouping). This highlights the benefits of whole genome sequence analysis compared to partial E gene sequence data analysis for TBEVs.

Our genome sequence represents the third complete genome from Kyrgyzstan and reveals the presence of at least two separate Siberian TBEV lineages in the region. The other two TBEV genomes from the region cluster in what is now recognised as the Bosnia lineage of TBEV-Sib, which also contains representative sequences from Bosnia, the Crimean peninsula and Kazakhstan. In contrast, the new genome clusters within the Vasilchenko lineage, suggesting a wider, more southern and western distribution for this lineage than was previously thought (Tkachev et al., 2020).

Our study highlights the importance of developing targeted next-generation sequencing strategies for TBEV to recover near-complete genome sequences from samples with low viraemia. The recovery of new complete TBEV genome sequences from endemic regions such as Kyrgyzstan will likely enhance our understanding of the true geographical range of different TBEV subtypes and lineages. This has important implications not only for clinics, given the observed differences in clinical manifestations between various TBEV subtypes, but also for molecular diagnostics, which should be optimised to detect circulating TBEV subtypes in different regions, and development of novel or improved TBEV vaccines.

## CRediT authorship contribution statement

**Jake D'Addiego:** Writing – original draft, Writing – review & editing, Methodology, Investigation, Data curation. **Mollie Curran-French:** Writing – review & editing, Investigation. **Jack Smith:** Writing – review & editing, Resources. **Asankadyr T Junushov:** Resources. **Irena Breininger:** Resources. **Barry Atkinson:** Writing – review & editing, Resources. **John Hay:** Project administration, Funding acquisition, Conceptualization. **Roger Hewson:** Writing – review & editing, Supervision, Project administration, Funding acquisition, Conceptualization.

## Declaration of competing interest

The authors declare that they have no known competing financial interests or personal relationships that could have appeared to influence the work reported in this paper.

## Data Availability

Data will be made available on request.

## Appendix Supplementary materials

Image, application 1

Image, application 2
