# Supplementary material for: Whole-genome sequencing surveillance of Siberian tick-borne encephalitis virus (TBEV) identifies an additional lineage in Kyrgyzstan
Source: Virus Res. 2024 Dec 22;351:199517. doi: 10.1016/j.virusres.2024.199517 (PMC11770319; doi:10.1016/j.virusres.2024.199517)
Supplement: Supplementary file 1 [file mmc1.zip › mmc1.docx]

**Supplementary Table S1.A – Primer pool 1**

| **Primer Name** | **Sequence (5’ – 3’)** | **Pool Concentration (µM)** |
| --- | --- | --- |
| TBEV_1_LEFT | GCTTCGGATAGCATTAGCAGCG | 2 |
| TBEV_1_LEFT_B | AGATTTTCTTGCACGTGCGTGCG | 2 |
| TBEV_1_RIGHT | TCCTTTCCTTCAGCCCTGATCA | 2 |
| TBEV_3_LEFT | ACGCATCTCACCAGAGTTGAAG | 2 |
| TBEV_3_RIGHT | CTGCCCTTTCCAAACAATCCG | 2 |
| TBEV_5_LEFT | AGCTTGACAAGACTCTGGAACAC | 2 |
| TBEV_5_RIGHT | TGTTCTCAATGGTGGGGTTTGG | 2 |
| TBEV_7_LEFT | CTGACAGTGATAGGAGAACACGC | 2 |
| TBEV_7_RIGHT | CGTGAGGTTTGCGTCTCCTT | 2 |
| TBEV_9_LEFT | TCAAGAATGGCATGGCGGTC | 2 |
| TBEV_9_RIGHT | TCACTGAGCAGCTCTCCGTT | 2 |
| TBEV_11_LEFT | TCGAGTTGGGGCCAGAGATT | 2 |
| TBEV_11_LEFT_B | TTGAGCTGGGACCAGAGATT | 2 |
| TBEV_11_LEFT_C | TCGAGTTGGGGCCAGAGATC | 2 |
| TBEV_11_RIGHT | CACGACAGTCAGTGGTTCACTAA | 2 |
| TBEV_13_LEFT | GAGAAAGAAGAGAGAGTGATGGC | 2 |
| TBEV_13_RIGHT | ATCCTTCTCCCTGTGTCCAAGA | 2 |
| TBEV_15_LEFT | AATGCATCGACAGACGCCTG | 2 |
| TBEV_15_RIGHT | CACGCTTTTCCCTTTTTGTCTCA | 2 |
| TBEV_17_LEFT | TGGAAGGAGGCGCAGATACT | 2 |
| TBEV_17_RIGHT | CTGCCAGGTTCCTCATGCAT | 2 |
| TBEV_19_LEFT | AGGCAGGAAAACAGAGAAGCAG | 2 |
| TBEV_19_RIGHT | TTGCCGAGAAGAAGACCTTGTG | 2 |
| TBEV_21_LEFT | ACTGTGGACAATGCCAGTGG | 2 |
| TBEV_21_RIGHT | GTGGTTGCATGCTGAACACATC | 2 |
| TBEV_23_LEFT | TCAACATCCAGTCAAGGAAACTCC | 2 |
| TBEV_23_RIGHT | GCTATGAATTCCTCTTTGCTGCAC | 2 |
| TBEV_25_LEFT | TGGAGCTGGAGTGGAAGGAATA | 2 |
| TBEV_25_RIGHT | ACCTGTCATCAATTGGTCTCACC | 2 |
| TBEV_27_LEFT | GACGGCCTGTCTTTCAAAAGC | 2 |
| TBEV_27_RIGHT | CTCCAGGTTTTGCTCTGTCACA | 2 |

**Supplementary Table S1.B – Primer pool 2**

| **Primer Name** | **Sequence (5’ – 3’)** | **Pool Concentration (µM)** |
| --- | --- | --- |
| TBEV_2_LEFT | GGTCAACAACAGACTGGATGGG | 2 |
| TBEV_2_RIGHT | GTCACCACACTTTCCACGGTCA | 2 |
| TBEV_2_RIGHT_B | GTCACCACACTTTCCACGGTTA | 2 |
| TBEV_4_LEFT | AAGCTATCGGACACCAAGGTTG | 2 |
| TBEV_4_RIGHT | TGTTTCCACGGTAGAGCCAG | 2 |
| TBEV_6_LEFT | TCTCTGGAACAAAACCCTGCAG | 2 |
| TBEV_6_RIGHT | CAACATCAGCACCCACTCCAA | 2 |
| TBEV_8_LEFT | TGGCATAGTACCACAAAACAGACT | 2 |
| TBEV_8_RIGHT | CTCAAGTCAGTGACCAATAGTTCAAC | 2 |
| TBEV_10_LEFT | AGTGGCAAGGTGATCCCGGA | 2 |
| TBEV_10_RIGHT | CCAGCTCCAAGACCAACAAGAG | 2 |
| TBEV_12_LEFT | TGTCCTGGATTGTCCCTTTGG | 2 |
| TBEV_12_LEFT_B | TGTCCTGGATTGTCCCTTTGA | 2 |
| TBEV_12_RIGHT | CTTCGGGCCGATCTCATCATTT | 2 |
| TBEV_14_LEFT | GGAGCCTGGAGGAAAAATGGAA | 2 |
| TBEV_14_RIGHT | AGTGAAACCTGACTCTCTTCCCA | 2 |
| TBEV_16_LEFT | GACTGGATCACCGAGTATGAAGG | 2 |
| TBEV_16_RIGHT | TCTGGCATCTTGTCCTGTTCTG | 2 |
| TBEV_18_LEFT | CATCAGAGAATTTGTGGCGTACG | 2 |
| TBEV_18_RIGHT | CTCAAGGAACCCCATCTCATTGG | 2 |
| TBEV_20_LEFT | CACGTGATGTCACTGGGAGT | 2 |
| TBEV_20_LEFT_B | CATGTAATGTCCCTGGGAGT | 2 |
| TBEV_20_RIGHT | TCCAGAGGTCACCAAGTGTGT | 2 |
| TBEV_22_LEFT | GCATACACAATTGGCGGAAAGG | 2 |
| TBEV_22_LEFT_B | GCATACACAATTGGCGGAAAAG | 2 |
| TBEV_22_RIGHT | AAAACCACGCACCGAGTTCC | 2 |
| TBEV_24_LEFT | CCTGGCACAAAGGTCATCATGA | 2 |
| TBEV_24_RIGHT | CCGTGTCATCCGCATAGAAGAG | 2 |
| TBEV_26_LEFT | CGATGGCTGAAAGAACATGGAGA | 2 |
| TBEV_26_RIGHT | CATGTCCTCTGTGGTCATCCA | 2 |
| TBEV_28_LEFT | GGTGGGGCCTGAAAAGTTTAGG | 2 |
| TBEV_28_LEFT_B | GATAGGACCTGAAAAATTTAAG | 2 |
| TBEV_28_LEFT_C | GAGCGGTGGAAAAGGTCAGG | 2 |
| TBEV_28_RIGHT | TCTGGGTGATGGTGGCTCAGG | 2 |
