# Supplementary figures and images for: Whole-genome sequencing surveillance of Siberian tick-borne encephalitis virus (TBEV) identifies an additional lineage in Kyrgyzstan
Source: Virus Res. 2024 Dec 22;351:199517. doi: 10.1016/j.virusres.2024.199517 (PMC11770319; doi:10.1016/j.virusres.2024.199517)

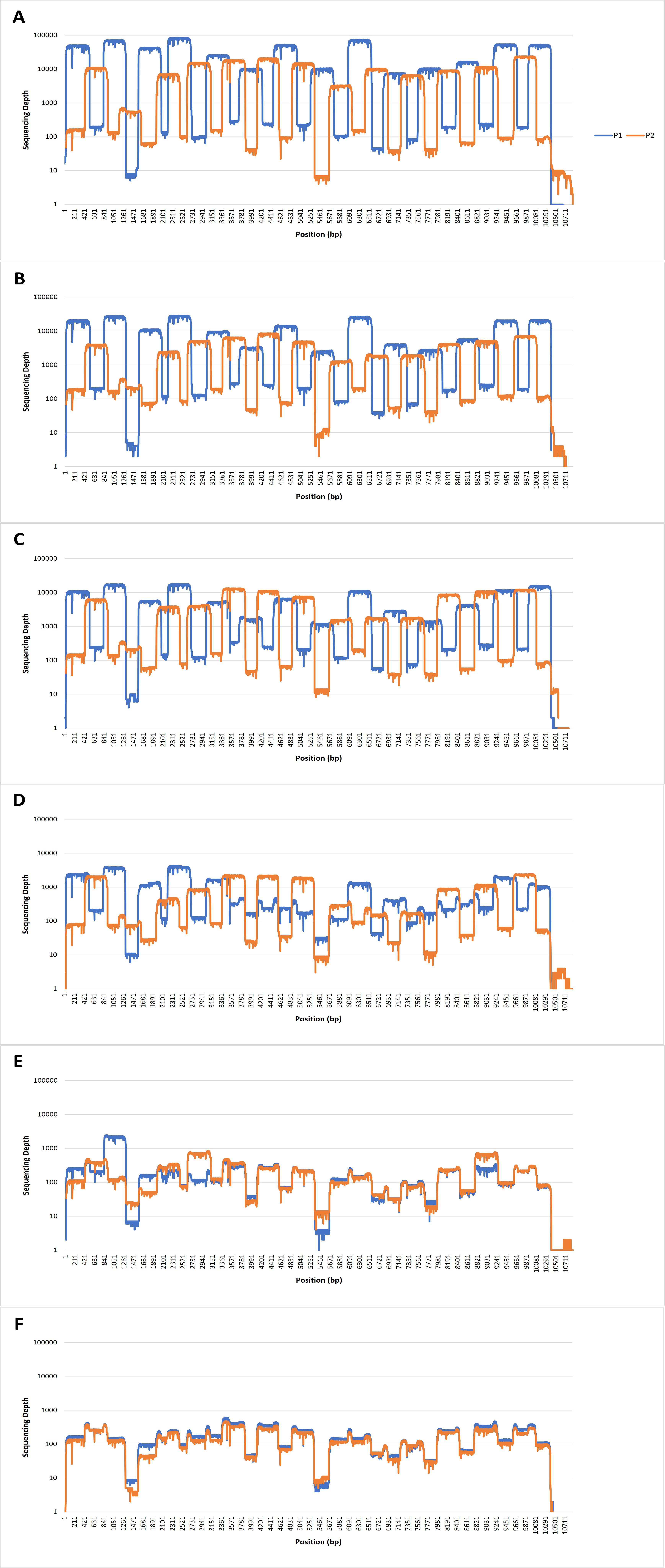

Supplement: Supplementary file 1 [file mmc1.zip › mmc2.jpg]
