# Supplementary material for: Whole-genome sequencing surveillance of Siberian tick-borne encephalitis virus (TBEV) identifies an additional lineage in Kyrgyzstan
Source: Virus Res. 2024 Dec 22;351:199517. doi: 10.1016/j.virusres.2024.199517 (PMC11770319; doi:10.1016/j.virusres.2024.199517)
Supplement: Supplementary file 2 [file mmc2.docx]

**Figure S1** – Sequencing depths and reference coverages for the serially diluted tick RNA samples. Samples with Ct values of 24.19, 27.82, 30.87, 34.28 and 37.7 are shown in graphs S1A-E. The NTC is shown in graph S1F. Primer pools 1 (P1) and 2 (P2) are represented respectively by the blue and orange lines.
